# A Multi-Static Radar Network with Ultra-Wideband Radio-Equipped Devices

**DOI:** 10.3390/s20061599

**Published:** 2020-03-13

**Authors:** Anton Ledergerber, Raffaello D’Andrea

**Affiliations:** Institute for Dynamic Systems and Control, ETH Zurich, 8092 Zurich, Switzerland

**Keywords:** network sensing, passive localization, device-free localization, RF-sensing, ultra-wideband, channel impulse response, IoT, multi-static radar, filtering

## Abstract

A growing number of devices, from car key fobs to mobile phones to WiFi-routers, are equipped with ultra-wideband radios. In the network formed by these devices, communicating modules often estimate the channel impulse response to employ a matched filter to decode transmitted data or to accurately time stamp incoming messages when estimating the time-of-flight for localization. This paper investigates how such measurements of the channel impulse response can be utilized to augment existing ultra-wideband communication and localization networks to a multi-static radar network. The approach is experimentally evaluated using off-the-shelf hardware and simple, distributed filtering, and shows that a tag-free human walking in the space equipped with ultra-wideband modules can be tracked in real time. This opens the door for various location-based smart home applications, ranging from smart audio and light systems to elderly monitoring and security systems.

## 1. Introduction

Ultra-wideband (UWB) technology is built into a growing number of devices. Larger companies are actively starting to invest in it [1,2]. Car key fobs equipped with UWB radios enable secure locking and unlocking of cars [3], phones equipped with UWB radios can share data based on their relative position [4], and WiFi-routers equipped with UWB radios offer an even larger range of localization-based services [5]. These applications are possible as UWB technology not only allows devices to communicate with each other, but also to localize with respect to each other. However, the devices are only aware of other UWB radio-equipped devices and need to rely on cameras or other sensors to sense and react to their environment.

**Figure 1:**
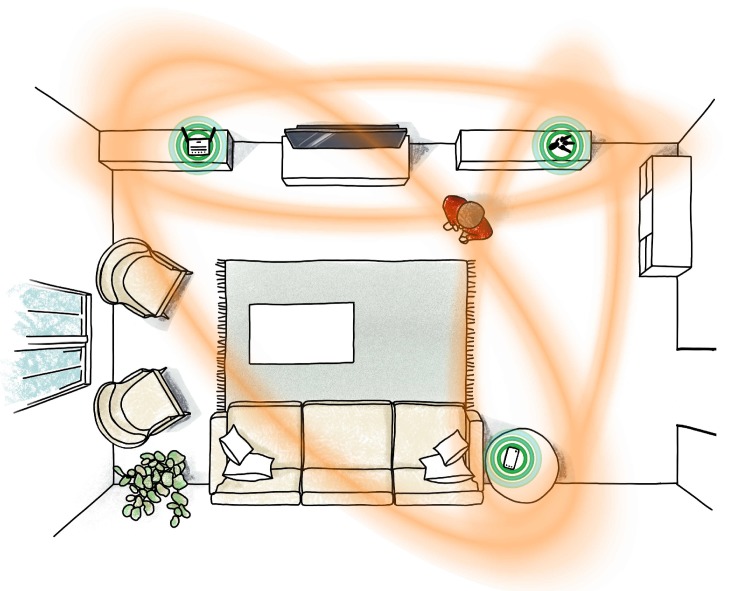
Three UWB radio-equipped devices forming a multi-static radar network for smart home applications. They can track movements in their surroundings, by detecting changes in the channel impulse responses of each communication channel and by intersecting the corresponding ellipses.

The work presented in this paper aims to augment UWB radio-equipped devices with the sensing capabilities of a multi-static radar network, thereby increasing their range of applications for entertainment purposes [6], elderly monitoring [7], security [8], or smart home applications in general (e.g., light and temperature control) [9]. In contrast to most existing UWB radar systems, which require specialized hardware, the system presented herein only relies on three or more communicating devices equipped with Decawave’s off-the-shelf, low-cost DWM1000 chip [10]. Figure 1 schematically shows how these devices can form a multi-static radar network and thereby track a nearby person,

1. by first localizing themselves with respect to each other,
2. by tracking changes in the channel impulse response of each communication channel,
3. by fusing these observed changes into a position estimate of a nearby person.

Since the presented signal processing algorithms are lightweight, they can be deployed in real time on devices with limited computational power, as for example found in Internet of Things (IoT) applications. The focus of this paper lies on step 2, but step 3 is also briefly discussed for evaluation purposes as experimental results are presented for the complete pipeline. For step 1, the reader is referred to [11] and the references therein. The dataset on which the experimental results presented are based is publicly available at [12]. See [13] or Supplementary Materials Video S2 for a video showing the system’s performance.

The remainder of this paper is structured as follows: Section 2 discusses related work. Section 3 introduces the necessary preliminaries on the channel impulse response (CIR), before its filtering is discussed in Section 4. The fusion of multiple filtered CIR to a position estimate of a tracked person is discussed in Section 5 and experimental results are presented in Section 6. Concluding remarks on the work, its limitations, and on future research directions are made in Section 7.

## 2. Related Work

Radio frequency (RF)-based, device-free localization has a long history, starting with the invention of the first radar systems in the early 20th century [14]. With the advent of low-power radio systems, human-centric, device-free localization recently started to emerge in indoor and IoT applications. For a general overview of these recent systems, the reader is referred to the survey papers [15,16], and, for an overview focusing on assisted living applications, the reader is referred to [17].

The work on human centric, device-free RF based localization can be split into systems where the transmitter and receiver are collocated, and into systems where they are separated. The former systems are known as monostatic radar systems, and usually have the transmitter and receiver driven by the same RF-clocks [18,19]. This rigid synchronization between the signal source and receiver enables accurate phase difference and correlation measurements of the transmitted and received signals. The latter systems are referred to as bi-static or multi-static radar systems and either combine multiple monostatic radars (as presented in [20,21]), or have the transmitters and receivers separated and driven by independent RF-clocks (as was done in [22]). In this case, they need to employ either optical fibres, wireless links, or early-late locked loops to synchronize their RF-clocks ([23], p. 293), which is also the case for the DWM1000 modules used in this work. Different transmitter and receiver positions or the measurement fusion of multiple distributed monostatic radars have the ability to improve radar performance as further discussed in [24].

Given the widespread use of WiFi-enabled devices, it is convenient to use their narrow band radio signals for localization. However, susceptibility to multi-path fading and the low temporal resolution make it hard to develop accurate tracking systems. Fingerprinting techniques help to improve the accuracy of such systems [25] but generally require labeled data gathering, which precludes fast deployment. An alternative approach to achieve a better accuracy is to increase the bandwidth by combining all available 5 GHz WiFi channels [26]. This is similar to systems which directly employ UWB signals for localization. Two frequently used UWB approaches are pulse-based UWB radars [22,23] and frequency-modulated continuous wave radars [18,19,27]. Both methods have their respective advantages [28], but, as pulse-based UWB is currently mainly used for device to device communication and active localization, it is convenient to extend such pulse-based UWB networks with the ability to sense their environment. This was also proposed in [22], where small temporal variations in the received UWB signals are utilized to localize a stationary human in a post-processing step. In addition, the 5G cell-phone networks utilize a large bandwidth, which makes them suitable for passive localization as well. First, experimental studies on passive localization using 5G networks to increase safety in vehicular environments are presented in [29,30]. Body proximity and motion detection have already proved feasible with previous generation cell-phone networks in [31].

Most of the work on UWB-based, human-centric, device-free localization is based on commercial, or custom-built, specific radar hardware. Therefore, these systems are able to achieve a high accuracy and resolution enabling them not only to track and identify multiple humans [19], but also to track them behind walls [32], and to remotely monitor their heart rate [33] and breathing frequency [34]. In contrast to employing these purposely built devices, researchers have also focused on how Decawave’s low cost, off-the-shelf UWB radios can be utilized for these applications, as also done in this work. For example, the authors in [35] demonstrated heart rate monitoring by analyzing the CIR between a DWM1000 radio placed on the chest and another placed on the back of a person. Passive localization using Decawave’s radios was investigated in [36]. By post-processing CIR measurements, they were able to detect a human in a corridor with up to 20 m distance to the UWB modules. The work presented in this paper can be seen as a significant progression of their work and previous work in as much as we track a human in real time by means of a self-localizing, multi-static radar network with off-the-shelf UWB radio modules. Unlike the systems discussed in [19,22], the method presented in this paper is not able to track multiple targets or to detect a static target. These limitations should be addressed in future work and would further close the gap to systems employing radar-specific hardware. Potential mitigation approaches are discussed in subsection of Section 7.

## 3. Channel Impulse Response

In UWB communication and localization systems, the channel impulse response between a transmitter and receiver is often estimated. It is used for matched filtering techniques to decode transmitted data [37,38], for accurately time-stamping received signals [39] and to establish a secure communication link between any two communicating modules [40]. In addition, in radar applications, the channel impulse response is of interest, as objects can be detected based on the trace they leave behind in the measured CIR, which will also be utilized in this work.

**Figure 2:**
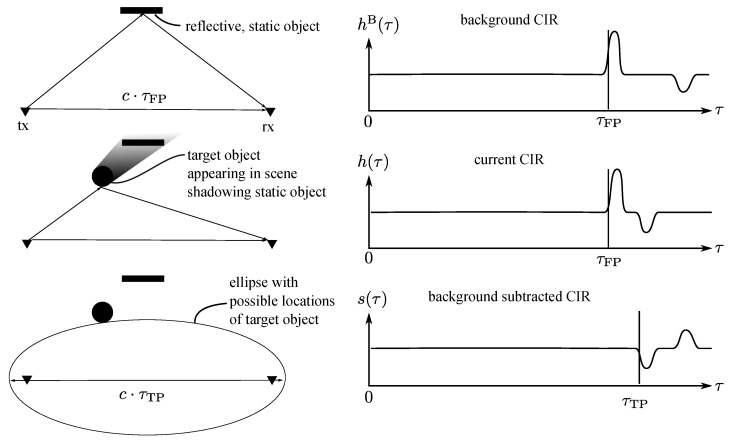
A visualization of the background subtraction technique, applied to localize a target object in the scene on an ellipse. First, the background CIR is acquired when no target object is present. The location of the first peak within this CIR corresponds to the time of flight of the signal taking the direct path and is denoted as $\tau_{\mathrm{FP}}$. This background CIR is then subtracted from the currently measured CIR. The location of the first peak within this background subtracted CIR corresponds to the time of flight of the signal reflected from the target and is denoted as $\tau_{\mathrm{TP}}$. It constrains the location of the target object to an ellipse, as defined in Equation (1).

A good overview of the CIR and UWB propagation channels is given in [41]. The CIR is informative of the different paths a signal takes when traveling from a transmitter to a receiver, as illustrated in Figure 2. Due to the short pulse duration of UWB signals, it is often possible to separate the first peak caused by the direct path from the peaks of other reflected and scattered paths. The location of first path $\tau_{\mathrm{FP}}$ within the CIR is generally found by means of a leading edge detection algorithm and allows accurate measurement of the time of flight.

If a new object appears in the scene, the newly measured CIR will differ from the previously measured CIR without the object. By subtracting the previous CIR measurement, termed background, from the current CIR measurement, and by applying a leading edge detection algorithm on the difference of the two measurements, the location within the CIR of the signal reflected from the object is found. This location is termed the target path location $\tau_{\mathrm{TP}}$ and constrains the position of the target object, $p_{T}$, to an ellipse in two dimensions. This ellipse has the position of the transmitter, $p_{\mathrm{tx}}$, and receiver, $p_{\mathrm{rx}}$, as foci points and a major axis of length $c\;\cdot \;\tau_{\mathrm{TP}}$, i.e.,

(1) $$\begin{array}{c} \left\|p_{\mathrm{tx}}-p_{T}\right\|+\left\|p_{\mathrm{rx}}-p_{T}\right\|=c\;\cdot \;\tau_{\mathrm{TP}} \end{array}$$

where we used *c* to denote the speed of light. This principle of subtracting the background from the current signal is termed “background subtraction” and is visualized in Figure 2. It is a widely used clutter removal technique in radar applications [42].

By having multiple transmitter and receiver pairs, multiple ellipses can be combined and the new object can be localized at the intersection point of these ellipses as visualized in Figure 1.

### CIR Measurements with Two DWM1000 Transceivers

In contrast to traditional mono-static radar systems, the transmitting and receiving radio modules in a UWB network are not triggered by the same RF-clock, but each have their own independent RF-clock running with slightly different and not always constant speed. Hence, CIR measurements between two such modules taken at different times generally also sample the CIR at different times. By aligning these different CIR measurements with the estimated first path location $\tau_{\mathrm{FP}}$, as outlined in [36], a high resolution estimate of the CIR can be obtained. Note that the DWM1000 modules do not directly estimate the CIR, but its complex envelope ([23], p. 281). However, for simplicity, we refer to the magnitude of this envelope simply as CIR in the following.

**Figure 3:**
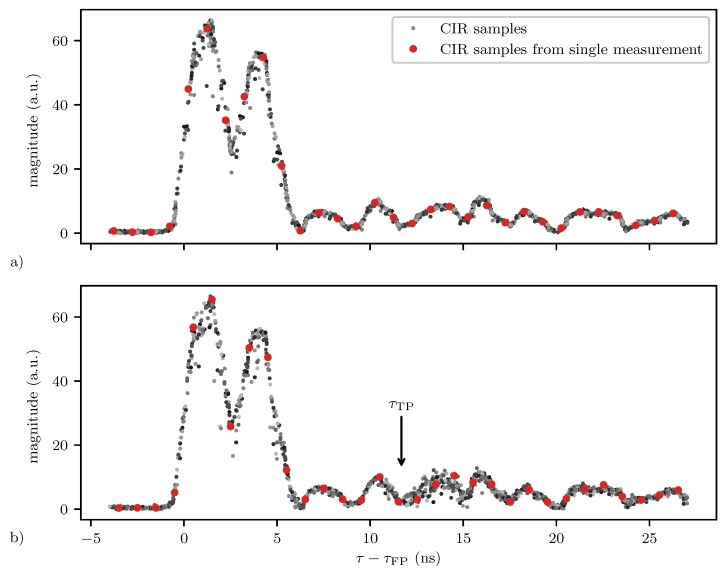
Plot (**a**) shows 50 measurements taken without a moving person in the vicinity of the UWB radios, i.e., in a static environment. Plot (**b**) again shows 50 measurements, now with a moving person. The black arrow marks the target path location $\tau_{\mathrm{TP}}$ within the CIR, i.e., the time of flight of the signal reflected from the target as calculated with ground truth measurements provided by a motion capture system, i.e., $\tau_{\mathrm{TP}}=\left(\left\|p_{\mathrm{tx}}-p_{T}\right\|+\left\|p_{\mathrm{rx}}-p_{T}\right\|\right)/c$. In both plots, a single CIR measurement is accentuated with red dots.

Plot (a) in Figure 3 shows such a high resolution, accumulated CIR consisting of 50 CIR measurements obtained with two DWM1000 within a time period Δt=27 ms in a static environment. The DWM1000 module provides CIR measurements with a resolution of approximately $\Delta \tau_{s}=1/\left(2f_{c}\right)\approx 1\;ns$, where $f_{c}=499.2\;M\mathrm{Hz}$ is the chipping frequency. Furthermore, it estimates the location of the first path $\tau_{\mathrm{FP}}$ within each CIR measurement with a resolution of $\frac{1}{64}\Delta \tau_{s}$ [43]. The samples of one such measurement are accentuated with red dots in Figure 3.

**Figure 4:**
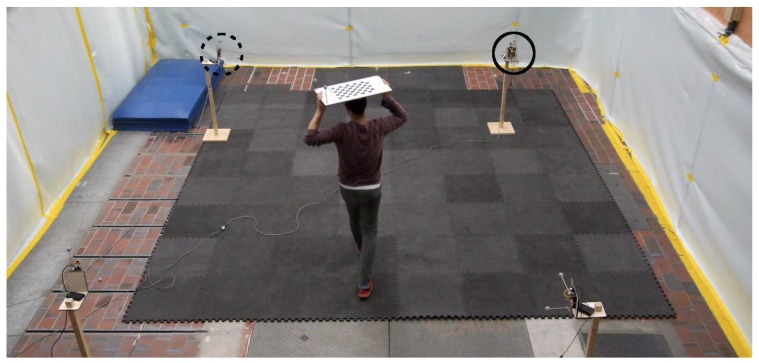
A person moving between four UWB radios is shown at the moment the CIR measurements shown in plot (b) of Figure 3 are obtained. The transmitting and receiving UWB radios of the CIR measurements shown in Figure 3 are marked with a solid and dashed circle, respectively. The board on top of the person’s head is for the overhead motion capture system with which ground truth measurements were obtained.

Plot (b) in Figure 3 shows the accumulated CIR for the same channel, but at a different time and with a person walking in the vicinity of the transmitting and receiving antenna as shown in Figure 4. It again consists of 50 CIR measurements, also obtained within a time period Δt=27 ms. While the mean signal of the accumulated CIR seems to be similar for the two plots, the variance of the accumulated CIR in the region $12\;ns\leq \tau -\tau_{\mathrm{FP}}<16\;ns$ is significantly larger in plot b). This is due to the person walking somewhere on the ellipse with a major axis of length $c\;\cdot \;\tau_{\mathrm{TP}}$ with $\tau_{\mathrm{TP}}\approx \tau_{\mathrm{FP}}+12\;ns$ and the receiver and transmitter as foci points. Therefore, the filtering algorithm presented in the following section is not based on the change in the mean (as illustrated in Figure 1), but on the change in the variance of the accumulated CIR.

## 4. CIR Filtering

It was shown in the previous section that a moving person in the scene leads to regions with a comparably higher variance in the accumulated CIR. The location of the first such region corresponds to the location of the target path $\tau_{\mathrm{TP}}$ used in Equation (1) to define the ellipse including the target position $p_{T}$. The goal of the following filtering algorithm is to extract the location of the target path $\tau_{\mathrm{TP}}$ from the CIR measurements.

Instead of transmitting the measured CIR to a central server and performing the filtering there, it is proposed to process the measured CIR on the host microcontroller of each DWM1000 module and to only transmit the extracted location of the target path. This frees up airtime due to the shorter package length, but also requires a lightweight filtering algorithm such that it can be run on the host microcontroller. The proposed recursive filtering approach is light both in terms of memory and computations. Note that more elaborate, finite impulse response or non-causal filtering approaches could improve the performance but were not investigated as they were not in line with our hardware and real-time constraints.

**Figure 5:**
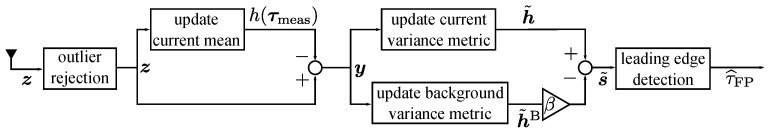
A signal-flow graph of the filtering algorithm proposed to extract the target path location ${\hat{\tau }}_{\mathrm{TP}}$ from CIR measurements z. The reader is referred to the main text body for a detailed explanation of the different blocks and signals.

A flowchart of the proposed algorithm is shown in Figure 5. Its individual steps are discussed in the following.

### 4.1. Mean Filtering

The proposed algorithm tracks the mean of the currently measured CIR in form of the coefficients $h=\left(h_{0},h_{1},\cdots ,h_{N_{\mathrm{knots}}-1}\right)$ of a uniformly-spaced, piece-wise linear function, i.e.,

(2) $$\begin{array}{c} h\left(\tau \right)=\left(1-\frac{\tau -\tau_{i}}{\Delta \tau_{\mathrm{knots}}}\right)h_{i}+\left(\frac{\tau -\tau_{i}}{\Delta \tau_{\mathrm{knots}}}\right)h_{i+1}, \end{array}$$

where *i* is such that $\tau_{i}\leq \tau <\tau_{i+1}$, and where the knots $\tau_{i}$ of the piece-wise linear parameterization are given as

(3) $$\begin{array}{c} \tau_{i}=\tau_{\mathrm{start}}+\Delta \tau_{\mathrm{knots}}\;\cdot \;i\;\mathrm{for}\;i=\left\{0,\cdots ,N_{\mathrm{knots}}-1\right\} \end{array}$$

with

(4) $$\begin{array}{c} N_{\mathrm{knots}}=\frac{\tau_{\mathrm{end}}-\tau_{\mathrm{start}}}{\Delta \tau_{\mathrm{knots}}}+1. \end{array}$$

The knot spacing $\Delta \tau_{\mathrm{knots}}$ is chosen such that it is a divisor of the CIR measurement sampling period $\Delta \tau_{s}$, i.e.,

(5) $$\begin{array}{c} \Delta \tau_{\mathrm{knots}}=\frac{\Delta \tau_{s}}{m}, \end{array}$$

where m∈{1,2,⋯,64}. The smallest spacing m=64 is given by the resolution of the DWM1000’s leading edge detection algorithm, which estimates the first path estimate location $\tau_{\mathrm{FP}}$ within the measured CIR with a resolution of $\frac{1}{64}\Delta \tau_{s}$. Such knot spacing reduces the number of necessary computations to update knot coefficients as explained in more detail in Appendix A. In order to have an estimate of the noise floor, $\tau_{\mathrm{start}}$ is chosen to be before the first path location, such that $h_{0}$ is indicative of the noise floor. Given the DW1000 settings as specified in Table A1, the CIR starts to rise approximately 1 ns before the estimated first path location as visible in Figure 3; hence, $\tau_{\mathrm{FP}}-\tau_{\mathrm{start}}$ must be larger than 1 ns. To add a safety margin, we choose $\tau_{\mathrm{FP}}-\tau_{\mathrm{start}}=4\;ns$.

Upon reception of a CIR measurement

(6) $$\begin{array}{cc} z & =\left(z_{0},z_{1},\cdots ,z_{N_{s}-1}\right) \end{array}$$

composed of *N*_s_ CIR samples with the corresponding sampling times

(7) $$\begin{array}{cc} \tau_{\mathrm{meas}} & =\left(\tau_{\mathrm{meas},0},\tau_{\mathrm{meas},1},\cdots ,\tau_{\mathrm{meas},N_{s}-1}\right) \end{array}$$

the innovation signal $y=\left(y_{0},y_{1},\cdots ,y_{N_{s}-1}\right)$ with respect to the current piecewise parameterization (Equation (2)) is calculated, i.e.,

(8) $$\begin{array}{c} y_{j}=z_{j}-h\left(\tau_{\mathrm{meas},j}\right)\;\mathrm{for}\;j=\left\{0,1,\cdots ,N_{s}-1\right\}. \end{array}$$

**Figure 6:**
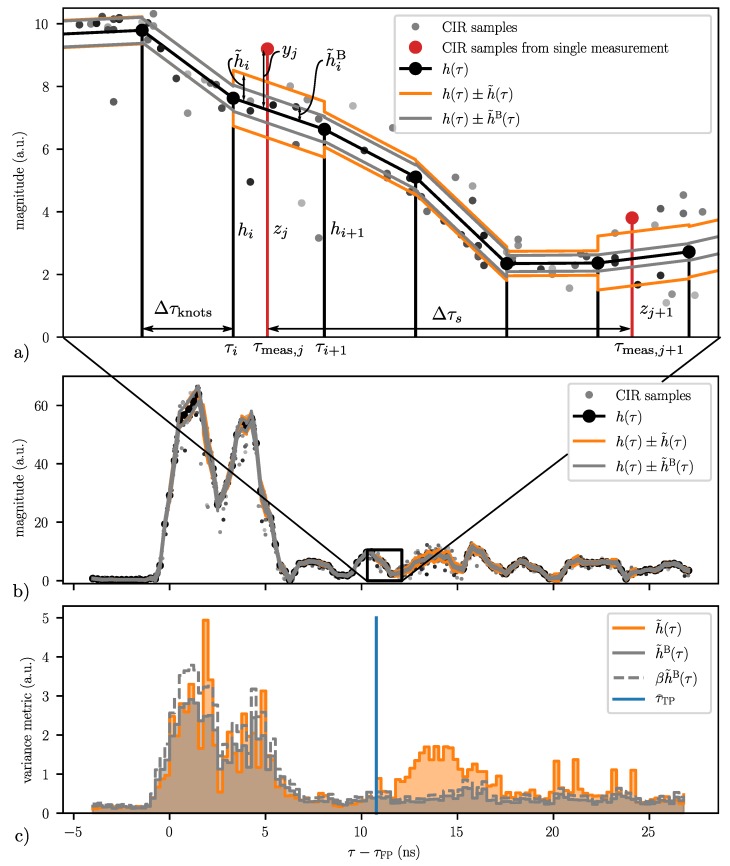
Plots (**a**) and (**b**) show the parametrization of the CIR at the time instance also corresponding to plot (**b**) of Figure 3 and Figure 4. Plot (**a**) shows an enlargement of the region marked with a black rectangle in plot (**b**). The piecewise-linear parametrization of the current mean is shown by the black lines, and the piecewise-constant parametrization of the current and background variance metrics are shown by the orange and gray lines, respectively. The black and red stems in plot (**a**) mark the knots and samples of a single CIR measurement, respectively. Plot (**c**) compares the current variance metric (orange) with the background variance metric (gray). The gray, dashed line is the background variance metric multiplied by the sensitivity factor as given in Equation (13). The blue line represents the the target path location as estimated by the leading edge detection algorithm given in Equation (14) with $N_{\mathrm{win}}=8$ and $N_{\mathrm{seg}}=5$.

This innovation signal is then used to recursively update the coefficients h

(9) $$\begin{array}{c} h\leftarrow h+Ky, \end{array}$$

where the gain matrix $K\in R^{N_{\mathrm{knots}}\times N_{s}}$ is further specified in Appendix A. A visualization of this parametrization is shown in Figure 6.

The coefficients h are initialized with the first received measurement z as

(10) $$\begin{array}{c} h_{i}\leftarrow \left\{\begin{array}{cc} z_{0}, & \;\mathrm{if}\;\tau_{i}<\tau_{\mathrm{meas},0}\\ z_{j}, & \;\mathrm{if}\;\tau_{\mathrm{meas},j}\leq \tau_{i}<\tau_{\mathrm{meas},j+1}\\ z_{N_{s}-1}, & \;\mathrm{if}\;\tau_{i}\geq \tau_{\mathrm{meas},N_{s}-1} \end{array}\right. \end{array}$$

for all $i\in \{0,1,\cdots ,N_{\mathrm{knots}}-1\}$, before they are updated according to Equation (9) with the consecutive measurements.

### 4.2. Innovation Signal Filtering

In addition to the mean, the proposed algorithm also tracks a variance metric of the currently measured CIR. In contrast to the mean, the variance metric is parameterized as a piecewise constant function with coefficients $\tilde{h}=\left({\tilde{h}}_{0},{\tilde{h}}_{1},\cdots ,{\tilde{h}}_{N_{\mathrm{knots}}-2}\right)$, which are updated according to

(11) $$\begin{array}{c} {\tilde{h}}_{l}\leftarrow {\tilde{h}}_{l}+\tilde{\alpha }(|y_{j}\left|-{\tilde{h}}_{l}\right) \end{array}$$

where $l\in \{0,1,\cdots ,N_{\mathrm{knots}}-2\}$ is such that $\tau_{l}\leq \tau_{j}<\tau_{l+1}$ and where the gain $\tilde{\alpha }\in \left(0,1\right)$. See Figure 6 for a visualization of the parametrization.

In order to distinguish variations in the tracked CIR from variations due to parameterization errors, system imperfections and environmental background noise, we track the background variation coefficients ${\tilde{h}}^{B}$ which are updated in the same way as $\tilde{h}$, i.e.,

(12) $$\begin{array}{c} {\tilde{h}}_{l}^{B}\leftarrow {\tilde{h}}_{l}^{B}+{\tilde{\alpha }}^{B}(|y_{j}\left|-{\tilde{h}}_{l}^{B}\right), \end{array}$$

but where ${\tilde{\alpha }}^{B}\ll \tilde{\alpha }$. All coefficients $\tilde{h}$ and ${\tilde{h}}^{B}$ are initialized with ${\tilde{h}}_{\mathrm{init}}$ (see Table 1 and Section 6.1 for parameter choices).

### 4.3. Background Subtraction and Leading Edge Detection

With the previously defined variance metrics for the currently measured CIR and the background CIR, regions in the CIR with temporarily higher variance can be detected by subtracting the background variance metric from the current variance metric

(13) $$\begin{array}{c} \tilde{s}=\tilde{h}-\beta {\tilde{h}}^{B}, \end{array}$$

where β>1 is a constant scalar sensitivity factor. Regions in the CIR with higher than usual variances are found in segments $\tau_{l}\leq \tau <\tau_{l+1}$, where *l* is such that ${\tilde{s}}_{l}>0$. The location of the target path $\tau_{\mathrm{TP}}$ is measured by finding the first cluster of such segments, i.e.,

(14) $$\begin{array}{c} {\hat{\tau }}_{\mathrm{TP}}=\min\tau_{l}\;s.t.\;{\tilde{s}}_{l}>0\;\mathrm{and}\;\sum_{n=l}^{l+N_{\mathrm{win}}}\left[{\tilde{s}}_{n}>0\right]\geq N_{\mathrm{seg}}, \end{array}$$

with $l\in \{0,1,\cdots ,N_{\mathrm{knots}}-2\}$, $\left[\;\cdot \;\right]$ the Iverson bracket converting a logical proposition into zero or one, $N_{\mathrm{win}}$ the window length, and $N_{\mathrm{seg}}$ the number of segments in the window which must have a higher than usual variance. This is visualized in plot (c) of Figure 6.

### 4.4. Outlier Rejection

An erroneous CIR measurement used to update the recursive filters for the mean of the CIR and variance metrics can corrupt the filtered signals and hence the measured target path location ${\hat{\tau }}_{\mathrm{TP}}$ for a significant amount of time. Therefore, CIR measurements are rejected if they do not fulfill the following criteria:

- A CIR measurement must be based on a minimal number of accumulated preamble symbols picked as half the number of preamble symbols transmitted (see Table A1 in Appendix B). We observed that a CIR measurement based on a lower number of preamble symbols is much more likely to correspond to an outlier.
- The CIR samples before the estimated first path location $\tau_{\mathrm{FP}}$ must be smaller than a multiple of the first knot coefficient $h_{0}$ which is indicative of the noise floor. A factor of five was used for the experimental results presented later. In case the estimated first path location $\tau_{\mathrm{FP}}$ is too late, CIR samples before the estimated first path are likely to be significantly higher than the noise floor.
- The largest sample of the measured CIR must not deviate too much from the maximal knot coefficient. In the experimental results presented, a measurement was rejected when its largest sample was more than five times smaller or two times larger than the maximal knot coefficient.

Many reasons can lead to such erroneous CIR measurements, such as the DWM1000’s leading edge detection algorithm failing to properly detect the first path location $\tau_{\mathrm{FP}}$ or packets colliding when two modules transmit at the same time, to name only a few. This outlier rejection strategy proved to be sufficient, but other, more elaborate strategies based on statistics could be devised.

## 5. Network Sensing

Section 3 briefly described how a moving person can be localized on an ellipse by tracking changes in the channel impulse response of one transmitter and receiver pair. If three or more UWB radio equipped devices track and broadcast such changes in the CIR, any device participating in the broadcasting or only listening to these messages can localize a person by intersecting the ellipses. To this end, it also needs to know the location of these devices.

### 5.1. Protocol

To enable such a sensing network, we commanded the devices to broadcast messages as given in Table 2. By listening to (and by broadcasting) these messages, a device can

- localize itself with respect to the other devices,
- synchronize its clock with respect to the network clock,
- localize a person within the space occupied by the network.

As already mentioned in the Introduction, we only discuss the last point and refer the reader to [44] for more details on the ALOHA protocol with which we scheduled message transmissions, and to [11,45] and the references therein for clock calibration and localization.

### 5.2. Particle Filter

The measured target path locations ${\hat{\tau }}_{\mathrm{TP}}$ contained in the network messages given in Table 2 can be fused in multiple ways to obtain the target position estimate ${\hat{p}}_{T}$. An analytical way is presented in [21] in which the intersection points of the different ellipses are calculated. Alternatively, the space of interest can be gridded and each cell can be assigned a probability that an object is moving within it (as described in [46]). Instead of spatially fixed cells, the probabilities can also be assigned to moving particles of a particle filter as done in this work (see [47] for an introduction to particle filtering). A random walk is assumed for the particles’ movements, i.e., each particle *p*’s position $p^{p}=\left(x^{p},y^{p}\right)$ is assumed to evolve as

(15) $$\begin{array}{c} x^{p}\left(t+\Delta t\right)=x^{p}\left(t\right)+\Delta t\;\cdot \;\eta_{x}^{p}\left(t\right) \end{array}$$

(16) $$\begin{array}{c} y^{p}\left(t+\Delta t\right)=y^{p}\left(t\right)+\Delta t\;\cdot \;\eta_{y}^{p}\left(t\right) \end{array}$$

where Δt is the time period since the last prediction and where $\eta_{x}^{p}\left(t\right),\eta_{y}^{p}\left(t\right)$ are drawn from a zero-mean, normal distribution with standard deviation of $\sigma_{\eta }$ at each prediction step. When a message is received, the particles’ weights are updated according to the likelihood conditioned on the measurement ${\hat{\tau }}_{\mathrm{TP}}$, i.e.,

(17) $$\begin{array}{c} w^{p}=p\left(\tau_{\mathrm{TP}}^{p}|{\hat{\tau }}_{\mathrm{TP}}\right), \end{array}$$

where we assume that the measurement error of ${\hat{\tau }}_{\mathrm{TP}}$ has Cauchy distribution centered at zero with scale parameter γ. This fat tail distribution was found to better represent the actual measurement error distribution than a normal distribution. The measured target path location ${\hat{\tau }}_{\mathrm{TP}}$ is contained in the message and the expected $\tau_{\mathrm{TP}}^{p}$ of each particle *p* is calculated according to Equation (1), i.e.,

(18) $$\begin{array}{c} \tau_{\mathrm{TP}}^{p}=\frac{1}{c}\left(\left\|p_{\mathrm{tx}}-p^{p}\right\|+\left\|p_{\mathrm{rx}}-p^{p}\right\|\right). \end{array}$$

Note that the position of the receiver $p_{\mathrm{rx}}$ and transmitter $p_{\mathrm{tx}}$ can be extracted from the current message and from previously received messages, respectively. Additionally, with every received message, a CIR measurement is obtained which can also be filtered and used to update the particle filter. After the weights of all $N_{P}$ particles have been calculated, the particles are resampled to get $N_{P}$ posterior particles, all with equal weights.

Note that this is a very basic approach. For improved performance, more elaborate measurement fusion approaches could be implemented, as, for example, the tailored particle filter outlined in [48].

## 6. Experimental Evaluation

In order to develop and test the algorithms before implementing them on the actual UWB radio equipped devices, a dataset of CIR measurements was recorded. The first part of this section discusses the dataset acquisition and tests the algorithms on it. Parameter settings are discussed which are also summarized in Table 1. The second part of this section discusses implementation details and real-time experimental results as also shown in the accompanying videos, see [13] or Supplementary Materials Video S2. In this paper, we focused on a lightweight filtering approach for devices with limited memory and computational power. However, to enable development of non-causal and more elaborate filtering techniques, we make the dataset available here [12].

### 6.1. Evaluation on CIR Measurements Dataset

Four devices equipped with DWM1000 modules connected to STM32F4 microprocessors were placed in a square with a side length of about 4 m, about 1.2 m above the ground, as shown in Figure 4. They were commanded to send messages to their peer modules at random intervals such that each device received messages with an average frequency of 564 Hz. In other words, each device received CIR measurements for each of its three communication channels with an average frequency of 188 Hz. Not the complete CIR, but only *N*_s_ =31 samples starting three samples before the estimated first path location were read from the DWM1000 register values. These samples were continuously sent via USB to a laptop which also logged ground truth measurements provided by an overhead motion capture system. During the first 17 s, the person inside the space was at rest and then started walking for 87 s. During the last 16 s, the person was at rest again. The settings employed by the DWM1000 are given in Appendix B, Table A1.

When applying the algorithms on this dataset, we look at the performance of the target path location filtering (described in Section 4) and at the performance of the particle filter, fusing the measured target path locations (described in Section 5.2). It is clear that the latter is heavily dependent on the first.

**Figure 7:**
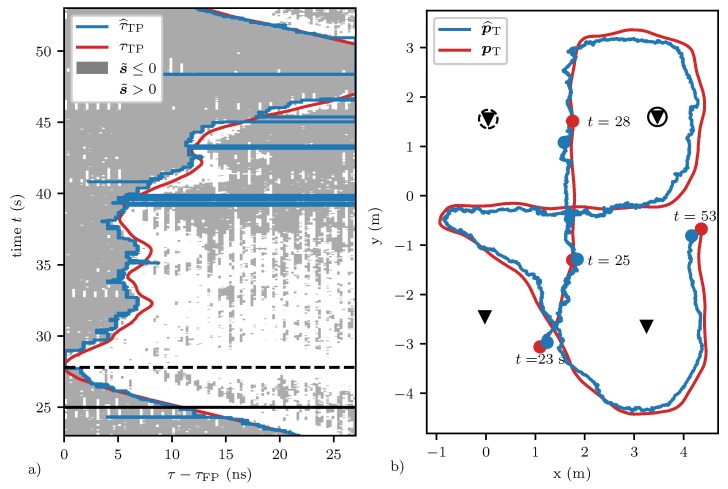
Plot (**a**) shows the measured and ground truth target path location $\tau_{\mathrm{TP}}$ in blue and red, respectively. The transmitter and receiver of the corresponding CIR are marked in plot (**b**) and also in Figure 4 with a dashed and solid circle, respectively. Plot (**b**) also shows the corresponding ground truth target position and the estimated target position in red and blue, respectively. The target path location is measured by performing a leading edge detection on the background subtracted variance metric $\tilde{s}$ (as defined in Equation (13)). Segments where $\tilde{s}$ is positive and negative are shown in plot (**a**) in white and gray, respectively. Note that sometimes the algorithm fails to detect the leading edge, e.g., at t≈40 s. The solid black horizontal line at t=25 s marks the time of the data shown in plot (**b**) of Figure 3, Figure 4 and Figure 6. The dashed black horizontal line at t=28 s marks the time when the target position was between the marked transmitter and receiver, i.e., when $\tau_{\mathrm{TP}}=\tau_{\mathrm{FP}}$.

Plot (a) in Figure 7 shows the measured target path location ${\hat{\tau }}_{\mathrm{TP}}$ (in blue) and the ground truth target path location (in red) as calculated with the data provided by the overhead motion capture system for the transmitter and receiver marked in plot (b) of the figure (as also marked in Figure 4). Plot (b) shows the corresponding target position trajectories provided by the motion capture system (in red) and by the particle filter (in blue), respectively. For better visibility, only a representative 30 s segment of the complete dataset is shown. Plot (a) also shows where the background subtracted variance metric $\tilde{s}$ (as defined in Equation (13)) is positive (white) and where it is negative (gray). This facilitates understanding the influence of the different parameters. For example, if the parameter $N_{\mathrm{seg}}$ utilized in Equation (14) is chosen too large with respect to $N_{\mathrm{win}}$, movement might not be detected, and, if it is chosen to be too small, noise or outliers might be detected. The sensitivity factor β has a similar effect. These parameters are kept constant at $N_{\mathrm{win}}=8$, $N_{\mathrm{seg}}=5$ and β=1.3 for the complete duration of the experiment. Environmental changes happening during the experiment can be dealt with to a certain extent, as we keep updating the background variance metric. Nevertheless, automatic parameter adjustment similar to the constant false alarm rate detection [49] might further increase performance. In addition, note that the measured target path location ${\hat{\tau }}_{\mathrm{TP}}$ tends to be smaller than the actual target path location in time periods when it is increasing. This effect can be explained by the recursive filtering approach used to estimate the variance metric of the CIR. Before the background subtracted variance $\tilde{s}$ can again become negative in a certain region, multiple CIR measurements with a low variance in this region need to be received. A higher value of $\tilde{\alpha }$ as given in Equation (11) helps to reduce this settling time, but also makes the algorithm more sensitive to noise. To speed up the initial convergence of the background variation coefficients ${\tilde{h}}^{B}$, the step size of the background variance metric, ${\tilde{\alpha }}^{B}$ as given in Equation (12), is initially set to ${\tilde{\alpha }}^{B}=0.1$ for quick convergence, and then set to ${\tilde{\alpha }}^{B}=0.001$ after having received a thousand CIR measurements. This number of measurements is received after about 5 s given the CIR measurement update frequency of 188 Hz for each communication channel.

The measured target path locations are fused using the particle filter outlined in Section 5.2. The initial position of the $N_{p}=200$ particles are randomly sampled from the region shown in plot (b) of Figure 7. The scale of the Cauchy distribution assumed for the measurement error is set to γ=1 ns and the variance of the process noise is set to $\sigma_{\eta }=10\;$m s^−1^. The positions of the devices are directly given by the motion capture system (see Section 6.2 for a results where the devices self-localize). The estimated position of the particle filter (as shown in blue in plot (b) of Figure 7) is taken as the particles’ mean position. The root-mean-squared error (RMSE) of the particle filter’s position estimate ${\hat{p}}_{T}$ was $\mathrm{RMSE}({\hat{p}}_{T})=0.35\;m$ when discarding the initial segment where the particles have not yet converged.

### 6.2. Implementation and Real-Time Results

The filtering algorithm discussed in Section 4, as well as the messaging protocol discussed in Section 5.1, were implemented on devices equipped with Decawave’s DWM1000 UWB radio modules and STM32F4 microprocessors (32-bit, 168 MHz, single-precision floating point uint, 196 kB RAM, 1 MB flash), which served as host microcontrollers to the UWB radios. These devices were placed in the experimental area, where they self-localized with respect to each other and where they measured and broadcasted changes in the CIRs.

An additional device only listened to network communication and forwarded it to a laptop running the particle filter described in Section 5.2. The same algorithms and parameters as used to evaluate the dataset in Section 6.1 were applied, with the difference that the particle filter only ran on the messages received by the additional device and used the devices’ positions as calculated from the self-localization algorithm instead of the ones provided by the motion capture system.

**Figure 8:**
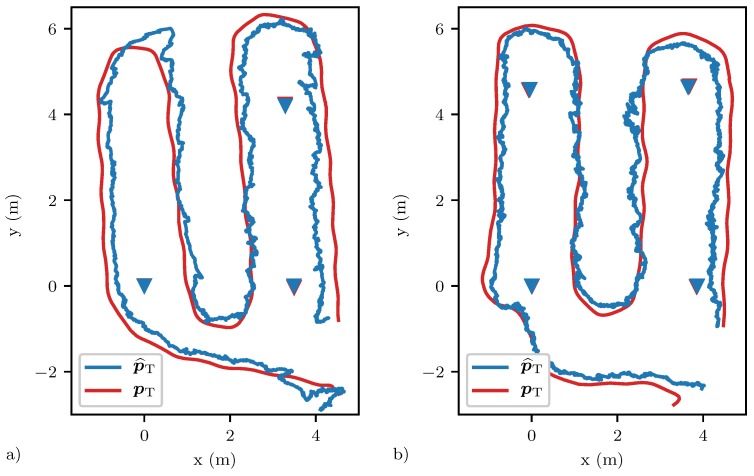
The real-time tracking performances of a network with three and four devices are shown in plots (**a**) and (**b**), respectively. The ground truth target positions and the estimated target positions are shown in red and blue, respectively. The estimated positions of the devices are shown with blue triangles with ground truth positions shown with red triangles beneath.

**Figure 9:**
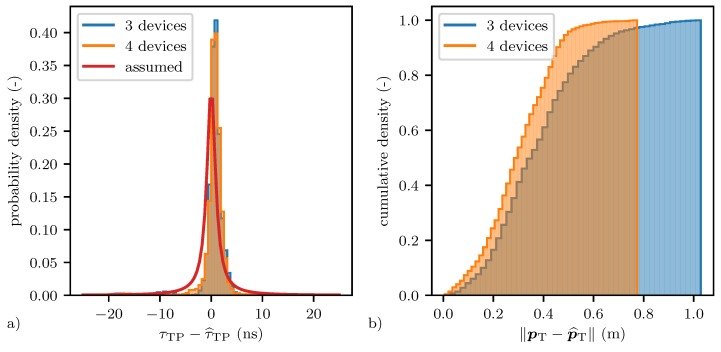
Plot (**a**) shows the measured target path ${\hat{\tau }}_{\mathrm{TP}}$ error distribution in blue and orange for experiments with three and four devices respectively, shown in Figure 8. The Cauchy distribution assumed for updating the particle filter is shown in red. Plot (**b**) shows the corresponding cumulative distributions of the error in the estimated target position estimates.

The particle filter’s position estimate for two such real-time experiments are shown in Figure 8, once for a network with three, and once for a network with four devices. The corresponding distributions of the error in the measured target path location ${\hat{\tau }}_{\mathrm{TP}}$ are shown in plot (a) of Figure 9. The Cauchy distribution assumed to update the particle filter with the measured target path location ${\hat{\tau }}_{\mathrm{TP}}$ is shown in red. The actual distributions which are shown in blue and orange both have a median of 0.8 ns. In addition, in other experiments, a median in between 0 ns and 1 ns was observed, which we assign to the recursive filtering approach as discussed in Section 6.1. The performance of the particle filter could be further improved by centering the Cauchy distribution at around 0.5 ns, which was however not investigated further. The particle filter’s position estimates for the network with three devices had an RMSE of $\mathrm{RMSE}({\hat{p}}_{T})=0.42\;m$, a median error of $\mathrm{MEDE}({\hat{p}}_{T})=0.36\;m$ and a maximum error of $\mathrm{MAXE}({\hat{p}}_{T})=1.03\;m$ when discarding the initial segment where the particles have not yet converged. For the network with four devices, these values were reduced to $\mathrm{RMSE}({\hat{p}}_{T})=0.33\;m$, $\mathrm{MEDE}({\hat{p}}_{T})=0.29\;m$ and $\mathrm{MAXE}({\hat{p}}_{T})=0.77\;m$. The cumulative error distribution of the two position estimates are shown in plot (b) of Figure 9. In order to calculate these metrics, the coordinate systems found by the devices had to be aligned with the coordinate system of the motion capture system. This was done by calculating the best-fitting rigid transformation that aligned the estimated and ground truth positions of the devices as outlined in [50]. These alignments are shown in Figure 8, where the red ground truth triangles marking the position of the devices are barely visible under the blue triangles representing the estimated positions. The error in the position estimates was on average only 3 cm, and therefore had little impact on the error in the target position estimate.

**Figure 10:**
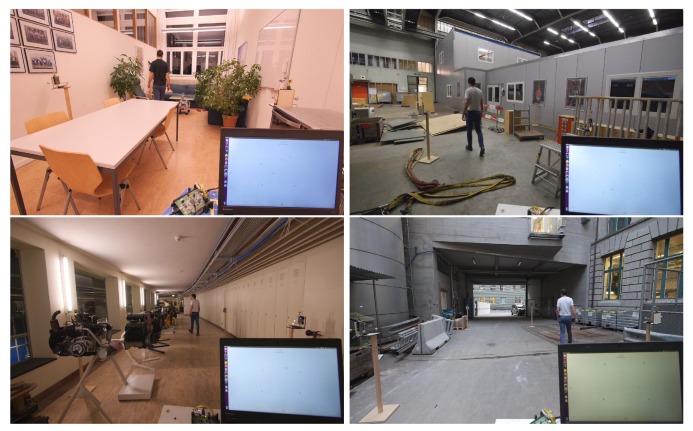
Four different environments in which the system is qualitatively evaluated. Footage of these real-time experiments is found in [13] or in the Supplementary Materials, Video S2.

Footage of such real-time experiments performed in the space shown in Figure 4 and in the environments shown in Figure 10 is found in the accompanying video, see [13] or Supplementary Materials Video S2. There it is visible that, even with plants obstructing the line-of-sight between the modules and a lot of metallic objects in the environment, the system is able to track a person with the background subtraction technique. However, if the metallic objects also obstructed the line-of-sight between the devices, the self-localization accuracy would be impaired. This would also result in an impairment in the target tracking performance, and would have to be circumvented by manually determining the devices’ locations with respect to each other.

## 7. Conclusions

This concept paper demonstrated that devices equipped with off-the-shelf DWM1000 UWB modules can form a multi-static radar network and track a moving person in their surroundings. The algorithms are deliberately kept simple and lightweight, such that they can be run on devices with limited computational power. This makes this approach interesting for location-based, IoT applications. However, performance can be improved by refining each of the presented steps. Additionally, the system can be further enhanced by addressing the following limitations.

### Limitations and Potential Mitigations

*Motion requirement:* The proposed system does not store or update the mean of the background, but only the background variance metric. Therefore, persons or objects which are not moving cannot be localized. By also storing the background mean and by performing leading edge detection on the background subtracted mean, this problem could be addressed. However, as we have seen in Figure 3, the mean might not vary much, making this a harder filtering problem when employing the DWM1000 instead of specialized hardware. Not only using the magnitude, but also the phase of the complex CIR envelope could help.

*Monitoring range:* We did not specify the monitoring range, but only recorded $\tau_{\mathrm{end}}-\tau_{\mathrm{FP}}=36\;ns$ of the CIR after the first path location. It is possible to read up to 1 μs (one symbol time) of the CIR from the DW1000 register values [51]. However, the power of a signal reflected from a far away object is low, resulting in a low signal to noise ratio. As this ratio is also heavily-dependent on the environment in which the measurements are made, it is difficult to indicate a meaningful monitoring range.

*Three-dimensional setup:* We only performed experiments with a two-dimensional setup. However, when four or more UWB radio-equipped devices are not all positioned in the same plane, it is also possible to localize an object in a three-dimensional setup at the intersection point of ellipsoids instead of ellipses as used in two dimensions.

*Multiple targets:* We only investigated tracking a single person. To track multiple people, the same algorithm to find the target path location within a CIR can be used, but a more evolved fusion algorithm than the simple particle filter outlined in Section 5.2 must be employed. The Cauchy distribution used for the measurement likelihood in this paper assumes a single target and is not suited for multiple targets moving in the scene. Instead, a multi-hypothesis tracking approach similar to the one proposed in [19] could be employed, where each target path location measurement would be assigned to a certain target position hypothesis. Other, particle filter-based, multi-target tracking approaches are discussed [52].

*Message Scheduling Protocol:* The ALOHA protocol employed allows easy handling of dynamic addition and removal of devices, and easy handling of partially connected networks with overlapping areas of coverage. However, it also leads to packet collisions, which is further discussed in [44]. A time-division multiple access (TDMA) protocol would prevent packet collision and allow for an increased CIR measurement update rate, which could further improve performance.

*Power consumption:* Contrary to what might be expected, the DWM1000 consumes most power ( 0.5 Watt) when in receive mode searching for preamble symbols and not when transmitting a message [53]. Therefore, it would be best if the UWB radios transmitted messages only at a high rate when a person is present and would only search for preamble symbols if a message is certain to arrive. A possible approach might be to only sound a single channel between two devices having a large battery and once a consistent target path location has been discovered, to wake up the other devices. Furthermore, with a TDMA protocol instead of an ALOHA protocol, the devices could limit the search for preambles to certain time periods in the TDMA protocol.

## Figures and Tables

**Table 1:** The parameters with which the experimental results presented in Section 6 were obtained. Note that in the dataset only $N_{s}=31$ CIR samples were logged, i.e., $\tau_{\mathrm{end}}-\tau_{\mathrm{FP}}=27\;ns$. In addition, note that the step size to update the background variance metric is set to ${\tilde{\alpha }}^{B}=0.1$ for the first thousand measurements, before being set to ${\tilde{\alpha }}^{B}=0.001$.

$\tau_{\mathrm{FP}}-\tau_{\mathrm{start}}$	$\tau_{\mathrm{end}}-\tau_{\mathrm{FP}}$	*m*	$\Delta \tau_{\mathrm{knots}}$	$N_{\mathrm{knots}}$	$N_{s}$	α	$\tilde{\alpha }$
4 ns	36 ns	4	0.25 n s	161	40	0.05	0.1
${\tilde{\alpha }}^{B}$	${\tilde{h}}_{\mathrm{init}}$	$N_{\mathrm{win}}$	$N_{\mathrm{seg}}$	β	$\sigma_{\eta }$	γ	$N_{p}$
0.001	4	8	5	1.3	10 m s^−1^	1 ns	200

**Table 2:** Content of broadcasted messages.

transmission time (in network time)
device ID
device position/location
device ID of transmitter of last received message
reception time of last received message (in network time)
target path location in CIR obtained with transmitter of last received message
other payload

## Supplementary Materials

The following are available online at https://www.mdpi.com/1424-8220/20/6/1599/s1, Video S1: A multi-static radar network with ultra-wideband radio-equipped devices (video abstract). Video S2: Real-time experiments as discussed in Section 6.2.

## Appendix A. Calculation of Coefficient Update Gain

The gain matrix $K\in R^{N_{\mathrm{knots}}\times N_{s}}$ could be chosen to be the gain of a Kalman filter with a first order random walk process model for the coefficients h and a measurement model according to Equation (2). Doing so would allow us to account for the initial uncertainty of the CIR shape by setting the initial covariance of the coefficients h to a large value. Furthermore, not all coefficients h are updated with the same rate because the estimated first path locations $\tau_{\mathrm{FP}}$ are not uniformly distributed (see [36] for more details). A Kalman filter could account for these different update rates. However, the necessary number of coefficients $N_{\mathrm{knots}}$ and hence the state dimension would be large, which is why such a Kalman filter was not investigated further, as it would not be suitable for running on microcontrollers.

Instead, sufficient performance was obtained with a gain matrix $K\in R^{N_{\mathrm{knots}}\times N_{s}}$ chosen such that a gradient descent step minimizing the quadratic loss

(A1) $$\begin{array}{c} \sum_{j=0}^{N_{s}-1}y_{j}^{2} \end{array}$$

was performed upon each reception of the channel impulse response. Consequently, the coefficients were updated as

(A2) $$\begin{array}{cc} h_{i} & \leftarrow h_{i}+\underset{K_{i,j}}{\underset{︸}{\alpha \left(1-\frac{\tau_{\mathrm{meas},\;j}-\tau_{i}}{\Delta \tau_{\mathrm{knots}}}\right)2}}y_{j} \end{array}$$

(A3) $$\begin{array}{cc} h_{i+1} & \leftarrow h_{i+1}+\underset{K_{i+1,j}}{\underset{︸}{\alpha \left(\frac{\tau_{\mathrm{meas},\;j}-\tau_{i}}{\Delta \tau_{\mathrm{knots}}}\right)2}}y_{j} \end{array}$$

for $j=\{0,1,\cdots ,N_{s}-1\}$ and where *i* is such that $\tau_{i}\leq \tau_{\mathrm{meas},j}<\tau_{i+1}$ and where α∈(0,1) denotes the gradient descent step size. Note that $\left(\tau_{\mathrm{meas},j}-\tau_{i}\right)\Delta \tau_{\mathrm{knots}}$ needs to be calculated only once for every measurement update as it is the same for all $\tau_{\mathrm{meas},j},\tau_{i}$-pairs if $\Delta \tau_{\mathrm{knots}}$ is chosen as proposed in Equation (5) (see also plot (a) in Figure 6).

## Appendix B. Parameter Settings

Table A1 shows the DWM1000 settings used for all experiments. These are default settings as specified in [51]. Performance is likely to change when employing other settings, but a systematic evaluation of the impact of the different settings is beyond the scope of this paper.

**Table A1:** The settings of the DWM1000 modules.

Channel Number	4 (Carrier Frequency 3993.6 MHz, Bandwith 900 MHz)
Pulse Repetition Frequency	16 MHz
Data Rate	6.8 Mbps
Preamble Length	128 Symbols
Preamble Accumulation Size	8
Preamble Code	7
Transmit Power Control	19 dB Gain
